# Pathogenic profiles and antimicrobial resistance in neutropenic acute leukemia patients: diagnostic potential of sTNF-R1 and IL-8

**DOI:** 10.3389/fonc.2026.1780095

**Published:** 2026-07-01

**Authors:** Yuan Zhang, Rong Wang, Guoqing Lv, Lihua Wang

**Affiliations:** 1 Department of Hematology, the First Affiliated Hospital of Henan Medical University, Weihui, China; 2 Key Laboratory for Leukemia Molecular Diagnosis and Treatment in Xinxiang City, Weihui, China; 3 Key Laboratory for Lymphoma Molecular Diagnosis and Treatment in Xinxiang City, Weihui, China

**Keywords:** acute leukemia, antimicrobial resistance, IL-8, infection, neutropenia, pathogen, sTNF-R1

## Abstract

**Objective:**

To analyze the etiological characteristics, antimicrobial resistance patterns of infections during the neutropenic phase in acute leukemia (AL) patients, and to evaluate the diagnostic value of soluble tumor necrosis factor receptor 1 (sTNF-R1) and interleukin-8 (IL-8).

**Methods:**

Clinical data from 142 AL patients in the neutropenic phase, admitted between July 2023 and July 2025, were retrospectively analyzed. Patients were divided into an infected group (n=63) and a non-infected group (n=79) according to the presence of hospital-acquired infections. Pathogen distribution and antimicrobial resistance were assessed in the infected group. Peripheral blood sTNF-R1 and IL-8 levels were measured within 24 hours after confirmed neutropenia and before antimicrobial therapy. Their diagnostic performance for concurrent infections was evaluated using receiver operating characteristic (ROC) curve analysis.

**Results:**

Hospital-acquired infections occurred in 63 patients (44.37%), with bloodstream infections (39.68%) and respiratory tract infections being the most frequent. A total of 71 non-duplicate bacterial strains were isolated, of which 45 (63.38%) were Gram-negative, predominantly Escherichia coli (14 strains, 19.72%), and 26 (36.62%) were Gram-positive, predominantly Staphylococcus aureus (10 strains, 14.08%). High resistance rates were observed to multiple antimicrobials, particularly ampicillin among Gram-negative bacteria and penicillin among Gram-positive bacteria. Compared with the non-infected group, the infected group had higher white blood cell counts, lower albumin levels, and a greater proportion of unfavorable cytogenetic prognosis (all P < 0.05). sTNF-R1 and IL-8 levels were significantly higher in the infected group (both P < 0.001). These biomarkers showed significant negative correlations with neutrophil and albumin levels, and positive correlations with white blood cell count (all P < 0.05). The areas under the ROC curve (AUC) were 0.903 for sTNF-R1 and 0.910 for IL-8 individually, increasing to 0.932 when combined (P < 0.001).

**Conclusion:**

Infections during the neutropenic phase in AL patients are predominantly caused by Gram-negative bacteria and exhibit substantial antimicrobial resistance. Elevated sTNF-R1 and IL-8 levels are associated with infection in this population. Combined detection of these biomarkers demonstrates good diagnostic performance and may serve as a useful tool for early screening of infections.

## Introduction

Acute leukemia (AL) is a highly aggressive hematologic malignancy characterized by the uncontrolled proliferation of immature blasts in the bone marrow and peripheral blood, resulting in severe suppression of normal hematopoiesis. Although chemotherapy remains the cornerstone of treatment for most patients with AL, it frequently induces profound myelosuppression, significantly increasing the risk of neutropenia (1). During the neutropenic phase, patients experience a marked impairment of innate immunity, rendering them highly susceptible to life-threatening infections. These infections not only prolong hospitalization but are also a leading cause of morbidity and mortality in this population (2).

In the setting of neutropenia, rapid and accurate diagnosis of infection remains a major clinical challenge. Inflammatory cytokines and their receptors play critical roles in the host response to infection. Soluble tumor necrosis factor receptor 1 (sTNF-R1), a key regulator of tumor necrosis factor-α (TNF-α) signaling, is closely associated with systemic inflammatory responses (3). Interleukin-8 (IL-8), a potent pro-inflammatory chemokine, promotes neutrophil chemotaxis and activation at sites of infection, thereby amplifying the inflammatory cascade (4). Emerging evidence suggests that sTNF-R1 and IL-8 may serve as valuable biomarkers for the early diagnosis and severity assessment of infections (5).

**Figure 1:**
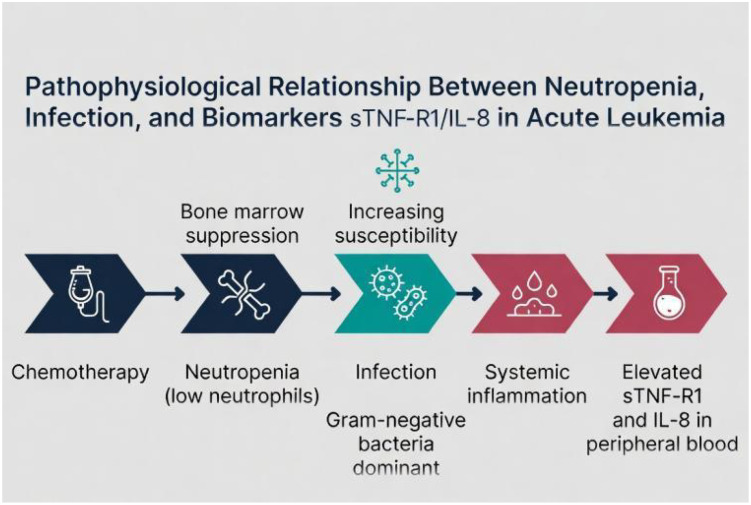
Flowchart graphic illustrating the pathophysiological links in acuteleukemia, showing chemotherapy causes bone marrow suppression and neutropenia, leading to infection from gram-negative bacteria, systemic inflammation, and elevatedsTNF-R1 and IL-8 in blood.

However, data regarding the diagnostic utility of sTNF-R1 and IL-8 specifically in AL patients during the neutropenic phase remain limited. Therefore, the present study aimed to investigate the etiological characteristics and antimicrobial resistance patterns of infections in AL patients during neutropenia, and to comprehensively evaluate the diagnostic performance of peripheral blood sTNF-R1 and IL-8 in this high-risk population. We hypothesized that these biomarkers could provide early and reliable indicators for infection detection, potentially facilitating timely clinical intervention. Building on this foundation, the present study aimed to investigate the etiological features of infections in AL patients during the neutropenic phase and to evaluate the diagnostic performance of sTNF-R1 and IL-8. The pathophysiological relationship between chemotherapy-induced neutropenia, infection, and the elevation of sTNF-R1 and IL-8 is illustrated in **Figure 1**.

## Materials and methods

### Study population

Clinical data from 142 patients with acute leukemia (AL) during the neutropenic phase, admitted to our hospital between July 2023 and July 2025, were retrospectively reviewed. Diagnostic criteria were as follows: (1) AL diagnosis (6, 7): peripheral blood or bone marrow blasts ≥20%, confirmed as acute lymphoblastic leukemia or acute myeloid leukemia by morphological and immunophenotypic analyses; (2) Neutropenia diagnosis (8): absolute neutrophil count <0.5 × 10^9^/L; (3) Hospital-acquired infection diagnosis (9): infections without a clear incubation period occurring more than 48 hours after admission, or infections with a defined incubation period occurring after the average incubation period post-admission.

Inclusion criteria were: (1) Confirmed diagnosis of AL receiving chemotherapy; (2) Age between 18 and 60 years; (3) Complete clinical records. Exclusion criteria included: (1) Active infection before enrollment; (2) Antibiotic use within one month prior to admission; (3) Concurrent autoimmune diseases or use of immunosuppressive agents; (4) Severe comorbidities or other malignancies.

### Detection methods

Specimens from suspected infection sites, including sputum, throat swabs, peripheral blood, bronchoalveolar lavage fluid, stool or anal swabs, and urine, were collected from patients in the infected group for microbiological culture, pathogen identification, and antimicrobial susceptibility testing, in strict accordance with the National Clinical Laboratory Procedures (10). The automated microbial identification system (bioMérieux, France) and corresponding reagents were used. Quality control strains (Staphylococcus aureus ATCC 25923, Pseudomonas aeruginosa ATCC 27853, Klebsiella pneumoniae ATCC 49247, and Escherichia coli ATCC 25922) were obtained from the National Center for Clinical Laboratories. Antimicrobial susceptibility results were interpreted according to the Clinical and Laboratory Standards Institute (CLSI) M100 guidelines (31st edition) (11).

To evaluate biomarker levels, 5 mL of fasting venous blood was collected from all enrolled patients within 24 hours after confirmed neutropenia and prior to the initiation of empirical antimicrobial therapy. Serum levels of soluble tumor necrosis factor receptor 1 (sTNF-R1) and interleukin-8 (IL-8) were measured using enzyme-linked immunosorbent assay (ELISA) kits (Shanghai Enzyme-linked Biotechnology Co., Ltd., batch numbers: ml038101 and ml103387).

### Outcome measures

(1) Pathogen distribution and antimicrobial resistance profiles in the infected group; (2) Clinical characteristics, including sex, age, AL subtype, body mass index (BMI), cytogenetic risk stratification, white blood cell count, absolute neutrophil count, platelet count, hemoglobin, albumin, smoking history, and alcohol consumption history; (3) Comparison of peripheral blood sTNF-R1 and IL-8 levels between the infected and non-infected groups.

### Statistical analysis

All statistical analyses were performed using SPSS software version 26.0. Normally distributed continuous variables are presented as mean ± standard deviation (SD) and were compared using independent samples t-tests. Categorical variables are expressed as frequencies and percentages [n (%)] and were compared using chi-square tests. Pearson correlation analysis was used to evaluate the associations between sTNF-R1/IL-8 levels and neutrophil count, white blood cell count, and albumin levels. Receiver operating characteristic (ROC) curves were constructed to assess the diagnostic value of sTNF-R1 and IL-8 for infections during the neutropenic phase in AL patients. A two-sided P-value <0.05 was considered statistically significant.

## Results

### Comparison of general characteristics between infected and non-infected groups

The baseline demographic and clinical characteristics of the infected group (n=63) and the non-infected group (n=79) are compared in **Table 1**. No statistically significant differences were observed between the two groups with respect to sex (χ² = 0.132, P = 0.717), age (40.60 ± 11.84 vs. 38.73 ± 12.13 years; t = 0.922, P = 0.385), AL subtype (χ² = 0.309, P = 0.578), body mass index (22.18 ± 1.52 vs. 22.12 ± 1.56; t = 0.196, P = 0.845), neutrophil count (0.27 ± 0.14 vs. 0.29 ± 0.12 ×10^9^/L; t = 0.646, P = 0.520), platelet count (44.98 ± 14.12 vs. 49.77 ± 14.79 ×10^9^/L; t = 1.947, P = 0.054), hemoglobin level (83.60 ± 15.82 vs. 87.31 ± 16.74 g/L; t = 1.342, P = 0.182), smoking history (χ ² = 0.294, P = 0.588), or alcohol consumption history (χ² = 0.503, P = 0.478).

**Table 1:** Comparison of general characteristics between infected and non-infected groups.

Characteristics	Infected (n=63)	Non-infected (n=79)	*χ*^2^/*t*	*P*
Sex				
Male	37 (58.73)	44 (55.70)	0.132	0.717
Female	26 (41.27)	35 (44.30)		
Age	40.60 ± 11.84	38.73 ± 12.13	0.922	0.385
AL types			0.309	0.578
Acute Lymphoblastic Leukemia	18 (28.57)	26 (32.91)		
Acute Myeloid Leukemia	45 (71.43)	53 (67.09)		
BMI	22.18 ± 1.52	22.12 ± 1.56	0.196	0.845
Cytogenetic Risk Stratification			4.322	0.038
Favorable Prognosis	34 (53.97)	56 (46.03)		
Adverse Prognosis	29 (70.89)	23 (55.77)		
White Blood Cell Count (×10^9^/L)	26.83 ± 5.64	24.82 ± 5.90	2.056	0.042
Neutrophil Count (×10^9^/L)	0.27 ± 0.14	0.29 ± 0.12	0.646	0.520
Platelet Count (×10^9^/L)	44.98 ± 14.12	49.77 ± 14.79	1.947	0.054
Hemoglobin (g/L)	83.60 ± 15.82	87.31 ± 16.74	1.342	0.182
Albumin (g/L)	30.52 ± 7.95	34.46 ± 8.15	2.880	0.004
Smoking History	12 (19.05)	18 (22.78)	0.294	0.588
Alcohol Consumption History	15 (23.81)	23 (29.11)	0.503	0.478

Significant differences were found in cytogenetic risk stratification (χ² = 4.322, P = 0.038), white blood cell count (26.83 ± 5.64 vs. 24.82 ± 5.90 ×10^9^/L; t = 2.056, P = 0.042), and serum albumin level (30.52 ± 7.95 vs. 34.46 ± 8.15 g/L; t = 2.880, P = 0.004). The infected group had a higher proportion of patients with unfavorable cytogenetic prognosis and higher white blood cell counts, along with lower albumin levels compared with the non-infected group (**Table 1**).

### Pathogenic bacterial distribution in infections during the neutropenic phase

A total of 71 non-duplicate bacterial strains were isolated from patients in the infected group. As shown in **Table 2**, Gram-negative bacteria were the predominant pathogens, accounting for 45 strains (63.38%), while Gram-positive bacteria comprised 26 strains (36.62%).

**Table 2:** Pathogenic bacterial distribution in infections during the neutropenic phase.

Pathogenic bacteria	Strains (n)	Percentage (%)
Gram-negative bacteria	45	63.38
Escherichia coli	14	19.72
Klebsiella pneumoniae	13	18.30
Pseudomonas aeruginosa	9	12.68
Acinetobacter baumannii	7	9.86
Other	2	2.82
Gram-positive bacteria	26	36.62
Staphylococcus aureus	10	14.08
Staphylococcus epidermidis	6	8.45
Streptococcus pneumoniae	5	7.04
Enterococcus faecium	3	4.22
Other	2	2.82

Among the Gram-negative isolates, Escherichia coli was the most common species (14 strains, 19.72%), followed by Klebsiella pneumoniae (13 strains, 18.30%), Pseudomonas aeruginosa (9 strains, 12.68%), and Acinetobacter baumannii (7 strains, 9.86%). Other Gram-negative bacteria accounted for 2 strains (2.82%).

For Gram-positive bacteria, Staphylococcus aureus was the most frequent isolate (10 strains, 14.08%), followed by Staphylococcus epidermidis (6 strains, 8.45%), Streptococcus pneumoniae (5 strains, 7.04%) (**Table 2**).

### Antimicrobial resistance profiles of major gram-negative pathogens in infections during the neutropenic phase

The antimicrobial resistance profiles of the major Gram-negative isolates are presented in **Table 3**. Escherichia coli (n=14) exhibited high resistance rates to ampicillin (92.86%), levofloxacin (71.42%), ciprofloxacin (78.57%), trimethoprim-sulfamethoxazole (71.42%), and gentamicin (57.14%). Resistance to carbapenems (imipenem and meropenem) was low (7.14%). Klebsiella pneumoniae (n=13) showed 100% resistance to ampicillin, with relatively lower resistance to fluoroquinolones and gentamicin. Resistance to carbapenems was also low (7.69%).Pseudomonas aeruginosa (n=9) demonstrated 100% resistance to ampicillin, while remaining relatively susceptible to several agents, including cefotaxime, cefoperazone/sulbactam, amikacin, and ciprofloxacin.Acinetobacter baumannii (n=7) exhibited the highest overall resistance among the four species, with resistance rates exceeding 50% to most tested antibiotics, including carbapenems (57.14%), cephalosporins, and gentamicin. Lower resistance was observed to amikacin (42.86%), levofloxacin (42.86%), and trimethoprim-sulfamethoxazole (42.86%) (**Table 3**).

**Table 3:** Antimicrobial resistance profiles of major gram-negative pathogens in infections during the neutropenic phase.

Antibacterial drugs	Escherichia coli (n=14)	Klebsiella pneumoniae (n=13)	Pseudomonas aeruginosa (n=9)	Acinetobacter baumannii (n=7)
Imipenem	1 (7.14)	1 (7.69)	1 (11.11)	4 (57.14)
Meropenem	1 (7.14)	1 (7.69)	1 (11.11)	4 (57.14)
Ampicillin	13 (92.86)	13 (100.00)	9 (100.00)	4 (57.14)
Ceftazidime	5 (35.71)	3 (23.08)	1 (11.11)	5 (71.42)
Cefotaxime	6 (42.86)	3 (23.08)	0 (0.00)	4 (57.14)
Cefoperazone/Sulbactam	2 (14.28)	2 (15.38)	0 (0.00)	4 (57.14)
Gentamicin	8 (57.14)	3 (23.08)	2 (22.22)	5 (71.42)
Amikacin	1 (7.14)	0 (0.00)	0 (0.00)	3 (42.86)
Levofloxacin	10 (71.42)	2 (15.38)	1 (11.11)	3 (42.86)
Ciprofloxacin	11 (78.57)	4 (30.76)	0 (0.00)	6 (85.71)
Sulfamethoxazole	10 (71.42)	5 (38.46)	1 (11.11)	3 (42.86)

### Antimicrobial resistance profiles of major gram-positive pathogens in infections during the neutropenic phase

The antimicrobial resistance profiles of the major Gram-positive isolates are summarized in **Table 4**. Staphylococcus aureus (n=10) exhibited high resistance to penicillin (90.00%) and moderate resistance to oxacillin (50.00%). Resistance rates to erythromycin, clindamycin, gentamicin, and fluoroquinolones were relatively low (20.00–40.00%). All isolates were susceptible to vancomycin, linezolid, rifampicin, and tetracycline. Staphylococcus epidermidis (n=6) showed complete resistance to penicillin (100.00%) and erythromycin (100.00%), with moderate resistance to clindamycin (33.33%) and oxacillin (33.33%). No resistance was observed to gentamicin, vancomycin, linezolid, rifampicin, or tetracycline. Streptococcus pneumoniae (n=5) demonstrated low resistance to penicillin (20.00%) and oxacillin (20.00%), but high resistance to erythromycin (80.00%) and clindamycin (80.00%). All isolates remained fully susceptible to vancomycin, linezolid, tetracycline, and fluoroquinolones.Notably, all Gram-positive isolates were susceptible to vancomycin and linezolid (**Table 4**).

**Table 4:** Antimicrobial resistance profiles of major gram-positive pathogens in infections during the neutropenic.

Antibacterial drugs	Staphylococcus aureus (n=10)	Staphylococcus epidermidis (n=6)	Streptococcus pneumoniae (n=5)
Penicillin	9 (90.00)	6 (100.00)	1 (20.00)
Erythromycin	4 (40.00)	6 (100.00)	4 (80.00)
Clindamycin	2 (20.00)	2 (33.33)	4 (80.00)
Benzylpenicillin	5 (50.00)	2 (33.33)	1 (20.00)
Gentamicin	2 (20.00)	0 (0.00)	2 (40.00)
Ciprofloxacin	2 (20.00)	1 (16.67)	0 (0.00)
Levofloxacin	2 (20.00)	1 (16.67)	0 (0.00)
Tetracycline	0 (0.00)	0 (0.00)	0 (0.00)
Rifampicin	0 (0.00)	0 (0.00)	0 (0.00)
Sulfamethoxazole	1 (10.00)	2 (33.33)	3 (60.00)
Linezolid	0 (0.00)	0 (0.00)	0 (0.00)
Vancomycin	0 (0.00)	0 (0.00)	0 (0.00)

### Comparison of peripheral blood sTNF-R1 and IL-8 levels between infected and non-infected groups

Peripheral blood levels of soluble tumor necrosis factor receptor 1 (sTNF-R1) and interleukin-8 (IL-8) in the infected group (n=63) and the non-infected group (n=79) are shown in **Table 5**. The infected group had significantly higher sTNF-R1 levels (3138.24 ± 1161.55 ng/L) compared with the non-infected group (1380.67 ± 561.69 ng/L; t = 11.026, P < 0.001). Similarly, IL-8 levels were significantly elevated in the infected group (455.80 ± 85.67 ng/L) than in the non-infected group (322.79 ± 44.79 ng/L; t = 11.166, P < 0.001) (**Table 5**).

**Table 5:** Comparison of peripheral blood sTNF-R1 and IL-8 levels between infected and non-infected groups.

Groups	n	sTNF-R1 (ng/L)	IL-8 (ng/L)
Infected	63	3138.24 ± 1161.55	455.80 ± 85.67
Non-Infected	79	1380.67 ± 561.69	322.79 ± 44.79
*t*		11.026	11.166
*P*		<0.001	<0.001

### Correlation analysis of peripheral blood sTNF-R1 and IL-8 levels with neutrophil count, white blood cell count, and albumin in infected AL patients during neutropenia

Correlations between peripheral blood sTNF-R1 and IL-8 levels and selected clinical parameters in the infected group are presented in **Table 6**. sTNF-R1 levels showed a significant negative correlation with neutrophil count (r = -0.532, P < 0.05) and albumin level (r = -0.406, P < 0.05), and a significant positive correlation with white blood cell count (r = 0.613, P < 0.05). Similarly, IL-8 levels were negatively correlated with neutrophil count (r = -0.579, P < 0.05) and albumin level (r = -0.311, P < 0.05), and positively correlated with white blood cell count (r = 0.621, P < 0.05) (**Table 6**).

**Table 6:** Correlation analysis of peripheral blood sTNF-R1 and IL-8 levels with neutrophil count, white blood cell count, and albumin in infected AL patients during neutropenia.

Parameter	sTNF-R1		IL-8	
	*r*	*P*	*r*	*P*
Neutrophils	-0.532	<0.05	-0.579	<0.05
White Blood Cell Count	0.613	<0.05	0.621	<0.05
Albumin	-0.406	<0.05	-0.311	<0.05

### Diagnostic value of peripheral blood sTNF-R1 and IL-8 for concurrent infections in AL patients during the neutropenic phase

**Figure 2:**
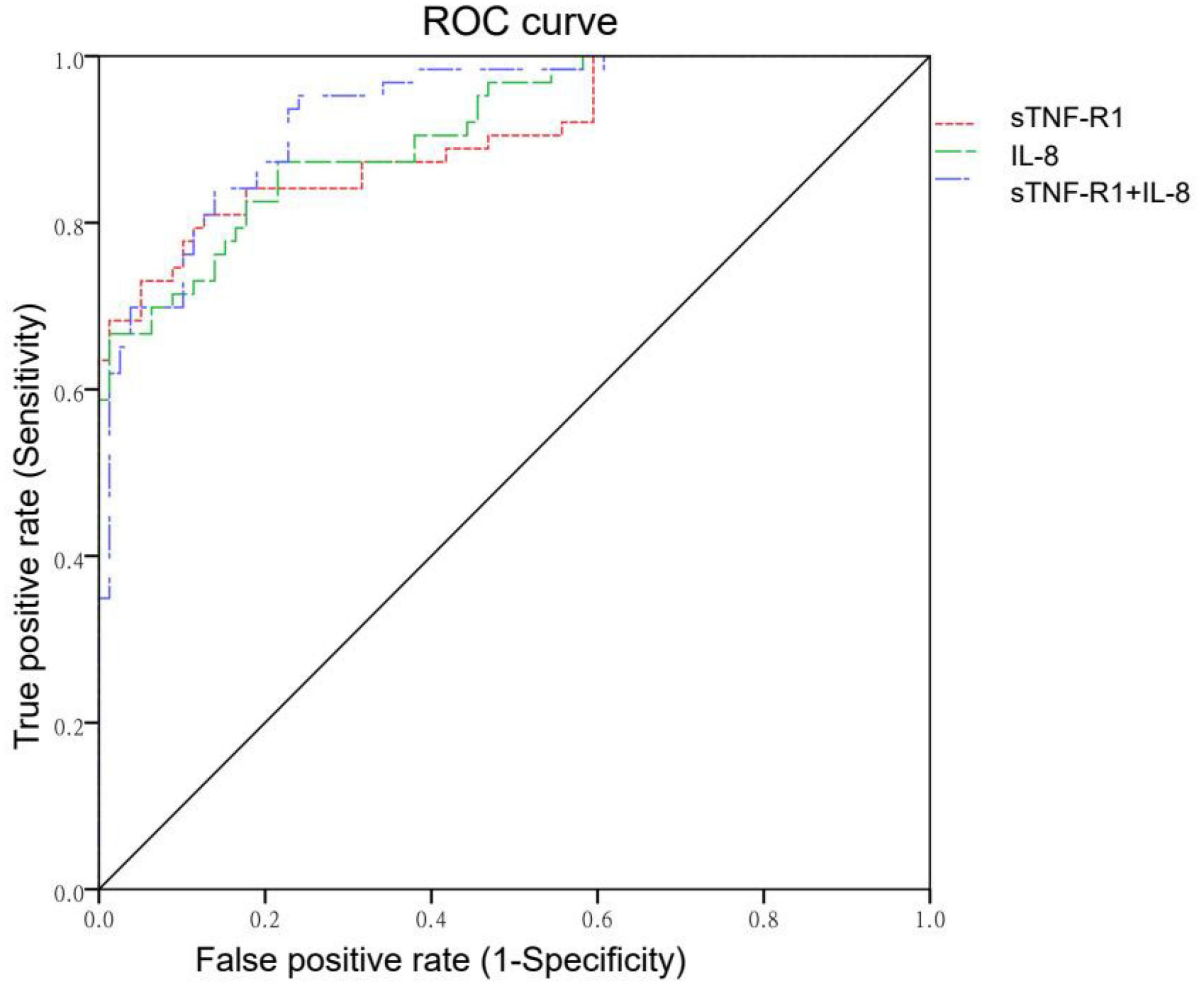
ROC curve comparing the diagnostic performance of sTNF-R1, IL-8, and the combination sTNF-R1 plus IL-8, showing sensitivity versus 1-specificity, with adiagonal reference line for random prediction.

Diagnostic Value of Peripheral Blood sTNF-R1 and IL-8 for Concurrent Infections in AL Patients During the Neutropenic PhaseReceiver operating characteristic (ROC) curve analysis was performed to evaluate the diagnostic performance of sTNF-R1 and IL-8 for detecting infections in AL patients during the neutropenic phase (**Table 7**). The area under the curve (AUC) for sTNF-R1 was 0.903 (95% CI: 0.851–0.954, P < 0.001), with an optimal cut-off value of 1980.31 ng/L, yielding a sensitivity of 80.95% and specificity of 87.34%. For IL-8, the AUC was 0.910 (95% CI: 0.863–0.956, P < 0.001), with a cut-off value of 352.75 ng/L, providing a sensitivity of 87.30% and specificity of 78.48%. When sTNF-R1 and IL-8 were used in combination, the AUC increased to 0.932 (95% CI: 0.894–0.971, P < 0.001), with a sensitivity of 95.24% and specificity of 75.95% (**Table 7**; **Figure 2**).

**Table 7:** Diagnostic value of peripheral blood sTNF-R1 and IL-8 for concurrent infections in AL patients during the neutropenic phase.

Item	AUC	Cut-off	sensitivity (%)	specificity (%)	*P*	95% CI
sTNF-R1	0.903	1980.31 ng/L	80.95	87.34	<0.001	0.851~0.954
IL-8	0.910	352.75 ng/L	87.30	78.48	<0.001	0.863~0.956
sTNF-R1+IL-8	0.932	/	95.24	75.95	<0.001	0.894~0.971

## Discussion

Chemotherapy-induced neutropenia in patients with acute leukemia (AL) markedly impairs innate immunity, resulting in a substantially increased risk of infections. In the present study, 63 patients (44.37%) developed hospital-acquired infections, with bloodstream infections (39.68%) and respiratory tract infections being the most common. Gram-negative bacteria accounted for 63.38% of the 71 isolated strains, with Escherichia coli (19.72%) as the predominant pathogen, followed by Gram-positive bacteria (36.62%), primarily Staphylococcus aureus. These findings are consistent with recent reports highlighting the continued dominance of Gram-negative pathogens in neutropenic fever among patients with hematological malignancies (11, 12).

Antimicrobial resistance analysis revealed high resistance rates among Gram-negative isolates to ampicillin, gentamicin, and several beta-lactams, as well as notable penicillin and oxacillin resistance among Gram-positive strains. These patterns align with current literature on the growing challenge of antimicrobial resistance in immunocompromised patients (13, 14), underscoring the need for local epidemiological surveillance and individualized antimicrobial therapy.

Infection triggers systemic inflammatory responses accompanied by elevated levels of soluble tumor necrosis factor receptor 1 (sTNF-R1) and interleukin-8 (IL-8). sTNF-R1 regulates TNF-α activity, while IL-8 is a potent chemokine that promotes neutrophil recruitment. In this study, both sTNF-R1 and IL-8 levels were significantly higher in the infected group than in the non-infected group. These findings are supported by previous studies that have linked these biomarkers with infection and clinical outcomes in cancer patients (15–17).

Correlation analyses demonstrated that sTNF-R1 and IL-8 levels were positively associated with white blood cell count and negatively associated with neutrophil count and albumin level in infected patients. Receiver operating characteristic (ROC) curve analysis showed AUC values of 0.903 for sTNF-R1 and 0.910 for IL-8 when used individually, with the combination achieving an AUC of 0.932. These results suggest that sTNF-R1 and IL-8, particularly in combination, may serve as useful early diagnostic markers for infections during the neutropenic phase in AL patients.

In summary, infections during the neutropenic phase in AL patients are predominantly caused by Gram-negative bacteria and are associated with substantial antimicrobial resistance. Elevated sTNF-R1 and IL-8 levels show promising diagnostic value, with their combination offering improved sensitivity for early detection.

### Limitations

This study has several limitations. First, it was retrospective and conducted at a single center with a relatively small sample size, which may limit the generalizability of the findings. Second, multivariable analyses adjusting for potential confounders were not performed due to sample size constraints. Third, external validation of the biomarker findings was not conducted. In addition, traditional inflammatory markers such as C-reactive protein (CRP) and procalcitonin (PCT) were not measured in this cohort, precluding direct comparison. Future multicenter, prospective studies with larger cohorts are warranted to validate these biomarkers and further evaluate their clinical utility relative to conventional markers.

## Data Availability

The datasets generated and/or analyzed during the current study are not publicly available due to ethical restrictions and patient privacy regulations but are available from the corresponding author upon reasonable request.
